# A Good View for Graph Contrastive Learning

**DOI:** 10.3390/e26030208

**Published:** 2024-02-27

**Authors:** Xueyuan Chen, Shangzhe Li

**Affiliations:** 1 State Key Laboratory of Software Development Environment, Beihang University, Beijing 100191, China; xueyuanchen@buaa.edu.cn; 2 School of Statistics and Mathematics, Central University of Finance and Economics, Beijing 100081, China

**Keywords:** graph contrastive learning, coding tree representation, structural entropy

## Abstract

Due to the success observed in deep neural networks with contrastive learning, there has been a notable surge in research interest in graph contrastive learning, primarily attributed to its superior performance in graphs with limited labeled data. Within contrastive learning, the selection of a “view” dictates the information captured by the representation, thereby influencing the model’s performance. However, assessing the quality of information in these views poses challenges, and determining what constitutes a good view remains unclear. This paper addresses this issue by establishing the definition of a good view through the application of graph information bottleneck and structural entropy theories. Based on theoretical insights, we introduce CtrlGCL, a novel method for achieving a beneficial view in graph contrastive learning through coding tree representation learning. Extensive experiments were conducted to ascertain the effectiveness of the proposed view in unsupervised and semi-supervised learning. In particular, our approach, via CtrlGCL-H, yields an average accuracy enhancement of 1.06% under unsupervised learning when compared to GCL. This improvement underscores the efficacy of our proposed method.

## 1. Introduction

Contrastive learning has demonstrated its effectiveness in various domains, including computer vision [1,2,3], natural language processing [4,5], and graph representation learning [6]. Specifically, in the context of graph representation learning, graph contrastive learning (GCL) [7] has proven to be a valuable approach. GCL boosts the performance of downstream tasks by pre-training a Graph Neural Network (GNN) on extensive datasets, often characterized by limited or absent annotations. This method has evolved into a practical self-supervised learning technique for effectively capturing graph representations.

In the realm of graph contrastive learning, two key modules have been delineated: graph augmentation and contrastive learning methodologies [8]. Similar to contrastive learning methods in other domains, those designed for graphs aim to enhance agreement among positive examples while minimizing it among negative samples. Graph augmentation employs diverse strategies such as node dropping, edge perturbation, attribute masking, and subgraph operations to generate augmented views [7]. Researchers have highlighted the pivotal role of view quality in the performance of contrastive learning models [9] and have focused on constructing effective views for graphs through data augmentation [10,11]. Unlike images, generating high-quality contrastive samples for graphs is challenging due to the intricate structural information embedded in graphical data [12]. This raises the following fundamental questions regarding how to address these challenges:

(1) *What defines a good view?*
(2) *What information should a good view include or exclude?*
(3) *How can a good view be generated?*

Recently, the information bottleneck (IB) theory has been applied to learn graph representation [13]. This has inspired the proposition that a good view for graph representation should possess minimal yet sufficient information, i.e., the essential information. Consequently, a metric for quantifying the quality of information embedded in graphs is indispensable. Taking cues from information theory, particularly the quantification of information in communication systems [14], researchers have grappled with the formidable task of measuring graph structural information, considered one of the “three great challenges for half-century-old computer science” [15]. Recently, the concept of structural entropy for graphs has been introduced to measure the uncertainty of graph structures, thus addressing this challenge [16]. This theory posits that minimizing uncertainty in a graph, or reducing its structural entropy, unveils the essential structure of the graph. In essence, a good view aims to minimize structural uncertainty, providing minimal yet sufficient information, and maximize benefits in graph contrastive learning with the least cost.

**Figure 1:**
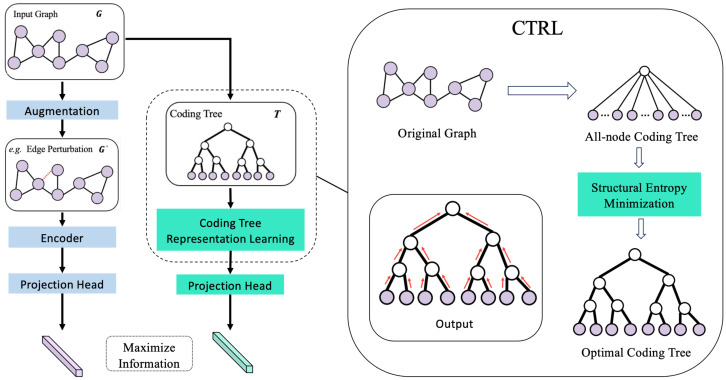
Framework. Our framework with the decoded essential graph structure for contrastive learning consists of two blocks. We adopt a view from previous works regarding graph contrastive learning. As for our good view, the original graphs are taken for coding tree transformation, and the representation for contrastive loss estimation can be obtained through the coding tree encoder.

In this research, we present a novel approach named CtrlGCL (refer to Figure 1) designed for graph contrastive learning, with a primary focus on the concept of a “good view” as defined earlier. Our methodology employs an optimization algorithm to decode essential structures by minimizing structural entropy. This decoding process generates coding trees, which represent essential structures corresponding to the given graphs. Subsequently, drawing inspiration from the message-passing mechanism inherent in Graph Neural Networks (GNNs), we propose an encoder tailored for learning representations from these coding trees, effectively capturing essential information. In comparison to existing effective views in previous studies, we conducted comprehensive experiments covering both semi-supervised and unsupervised learning across various graph classification benchmarks. The results demonstrate superior performance compared to state-of-the-art (SOTA) methods. The contributions are as follows:

- We are the first, to the best of our knowledge, to formulate a definition for a “good view” in the context of graph contrastive learning, grounded in the theories of graph information bottleneck and structural entropy.
- Drawing on these theoretical insights, we introduce CtrlGCL as a method to actualize the concept of a good view for graph contrastive learning, employing coding tree representation learning.
- Our proposed methodology for constructing good views was comprehensively assessed across various benchmarks, encompassing unsupervised and semi-supervised learning scenarios. The results consistently showcase its superior performance compared to state-of-the-art methods, underscoring the efficacy of our approach.
  In this article, the initial section provides a comprehensive overview of the background and outlines our specific contributions. The subsequent section delves into the existing research on graph contrast learning and structural entropy. Following that, the third section elucidates our theory and delineates the instantiation of our model. Moving forward, the fourth and fifth sections expound upon the experimental setup and present the obtained results. Finally, the concluding section encapsulates the essence of our study, providing a succinct summary of our findings.

## 2. Related Work

### 2.1. Graph Contrastive Learning

In the wake of the success achieved by contrastive learning in Convolutional Neural Networks (CNNs) for unsupervised image representation learning [2,9], the application of contrastive learning for graph representation learning has gained traction due to the scarcity of labels in real-world network data [7,10,11,12,17]. However, unlike image data augmentation, which does not demand extensive domain knowledge, augmentation in graph data is more intricate and challenging to analyze, posing difficulties in generating high-quality contrast samples [12,17,18]. Thus, the investigation of the contrastive view becomes a pivotal aspect of graph contrastive learning. Initially, contrastive pairs were constructed from different graph components, forming diverse contrastive modes [6,19,20,21]. More recently, inspired by the heuristics view design in computer vision, GCL introduced four types of views with random augmentation [7]. However, the optimal view combinations needed extensive evaluation. Subsequent works, like JOAO, proposed a search strategy based on the Min–Max principle for efficient view selection [11]. Similarly, AD-GCL aimed to produce graph views through learnable edge dropping [10]. Furthermore, LP-Info introduced a view-producing model via graph generation to avoid prefabricated data augmentations that required domain knowledge [17]. In addition to these foundational studies on GCL, an increasing number of researchers have employed the GCL methodology for recommendation systems. Specifically, CGI [22] adheres to the design principles of AD-GCL, learning to determine whether to drop an edge or node under the guidance of the information bottleneck principle. On the other hand, LightGCL [23] opts for graph reconstruction, leveraging singular-value decomposition for contrastive augmentation.

Despite the effectiveness of these graph views on various tasks, the data augmentations proposed, such as random perturbation, in existing methods may introduce structural damage and noisy information [7,10]. Similarly, learnable views through graph generation (i.e., LP-Info and LightGCL), based on different experimental settings, may not prevent artificially introduced noise. Additionally, methods like AD-GCL and CGI, relying on forced edge dropping, might suffer from graph structure damage and yield an undesirable performance in various regularizing settings [10]. In contrast, CtrlGCL provides theoretical guiding principles for contrastive view generation via an optimization algorithm that avoids random corruption and artificially introduced noisy information.

### 2.2. Structural Entropy

The need to measure information in communication networks gave rise to information entropy [14]. Several metrics have been developed for quantifying information in graphs. p(G) may be used to evaluate the entropy of graphs worldwide [24]. Different methods attempt to quantify the structural entropy of nodes in a signal network. Based on distance, the first example of local graph entropy measurement was presented [25]. Subsequently, numerous research projects were undertaken in an effort to quantify a graph’s structural information from various angles. These projects included Gibbs entropy [26], parametric graph entropy [27], and von Neumann entropy [28]. However, these definitions all destructure the graph into an unstructured probability distribution and then apply Shannon entropy to define the information of the graph. Therefore, these metrics do not suit the measurement of structural information, which is crucial for graphs and the key to the success of GNNs. In addition, these graph entropy definitions are only statistical mechanics approaches, providing an approach to comparing the different models of networks rather than an approach to figure out the minimal structural entropy of a given graph.

In more recent work, structural entropy was introduced and applied to evaluate the hierarchical structure complexity in a graph [16]. It was based on coding trees. Structural entropy was further established and used for decoding the fundamental graph structure, with an emphasis on measuring graph information through fixed hierarchical structures [29].

## 3. Materials and Methods

### 3.1. Preliminaries

Here, we introduce some preliminary concepts and notations. In this study, given a set of graphs $G=G_{1},G_{2},\cdots ,G_{M}$, every graph can be written as G=(V,E), where V and E are the sets of nodes and edges, respectively. The graph *G* may have node attributes $X_{V}=X_{v}|v\in V$.

#### 3.1.1. Graph Representation Learning

Graph Neural Networks (GNNs) with a message-passing method were used as encoders in this work. The purpose of GNNs is to learn a vector $h_{G}\in R$ for the whole graph *G* and an embedding vector $h_{v}\in R$ for each node. The initial node representation $h_{v}$ is updated iteratively using the GNN, starting at $h_{v}^{\left(0\right)}=X_{v}$. When an L-layer GNN is used, each node representation’s update takes into account data from the nodes that are nearby within L hops. According to Gilmer et al. [30], the L-layer of a GNN may be written as follows:

(1) $$h_{v}^{\left(l\right)}=f_{U}^{l}\left(h_{v}^{\left(l-1\right)},f_{M}^{\left(l\right)}\left(\left(h_{v}^{\left(l-1\right)},h_{u}^{\left(l-1\right)}\right)|u\in N\left(v\right)\right)\right),$$

where *h* is the node representation of *v* in the L-th layer, N(v) is the neighborhood node set for node *v*, $f_{U}^{l}$ is the update function in the L-th layer, and $f_{M}^{l}$ is the trainable message-passing function in the L-th layer. The node representation $h_{v}$ is similar to a subgraph in that it is a summary of the nearby nodes. As a result, the whole-graph representation is formalized as follows after L iterations:

(2) $$h_{G}=f_{R}\left(h_{v}|v\in V\right),$$

where the readout function that pools the final collection of node representations is denoted by $f_{R}$.

#### 3.1.2. The Mutual Information Maximization

Graph contrastive learning operates under the mutual information maximization (InfoMax) principle [20], where the objective is to maximize the degree of correspondence between a graph’s representations and various augmented perspectives. The goal of the graph representation $h_{G}$ is to capture the unique characteristics of the graph *G* such that the representation can effectively distinguish this graph from others. The following is how mutual information maximization aims to be expressed:

(3) $$\mathrm{InfoMax}:\;\;\;\;\max I\left(G;h_{G}\right),\;\mathrm{where}\;G∼P_{G},$$

where I(·) signifies the mutual information between two random variables, and $P_{G}$ represents the distribution defined over the graph *G*.

#### 3.1.3. Methodology

In this section, we begin by outlining our theoretical reasoning and then attempt to provide a definition of a good perspective. We next offer a particular instantiation of the good view tailored for graph contrastive learning, building upon structural information theory.

### 3.2. The Essential Structure with Minimal Structural Uncertainty

Given the challenge of limited labeled data in real-world graph datasets, obtaining meaningful representations and establishing effective pre-training is contingent upon self-supervised models delving into the intrinsic information of graphs [31]. The choice of “views” in self-supervised learning becomes pivotal as it dictates the information encapsulated by the model’s representation [9]. While there have been prior works addressing the design of effective views for graph contrastive learning from various perspectives [7,10], none of them offer a clear definition of a good view aligned with the fundamental objective stated above. Our investigation seeks to fill this gap by providing a precise definition of what constitutes a good view in the context of graph contrastive learning.

In the realm of computer vision, researchers have provided an empirical solution, suggesting that a good view involves compressing the mutual information between views while preserving information relevant to downstream tasks [9]. The notion of the information bottleneck, or more precisely the graph information bottleneck (GIB), has been presented in relation to Graph Neural Networks (GNNs) [13]. A similar approach is put out by GIB, which drives our investigation into what makes a good perspective for graph contrastive learning. Models can obtain minimum yet adequate information for a particular task by simultaneously minimizing mutual information between the input and the output (i.e., minI(G;f(G))) and maximizing it between the model’s output and the target (i.e., maxI(f(G);Y)). The following is how the GIB aim is stated:

(4) $$\mathrm{GIB}:\;\;\;\;\max_{f}I\left(f\left(G\right);Y\right)-\beta I\left(G;f\left(G\right)\right),$$

where β is a positive constant and $\left(G,Y\right)∼P_{G\times Y}$. We suggest that, under the framework of GIB, an optimal perspective for graph contrastive learning should capture the least amount of information necessary for tasks that come after, hence optimizing gains at the lowest possible expense.

It is undoubtedly important to note that the first part of GIB needs target-specific data for the job at hand (that is, *Y*), which presents difficulties for the self-supervised training framework. This challenge, however, directs our attention to the latter portion of GIB, which is independent of such target-related data. This feature clarifies the investigation of what, in the context of graph contrastive learning, makes a good view. Moreover, the goal of decreasing the mutual information between the input graph and the learnt representation (that is, minI(G;f(G))) emphasizes the essential information that graph contrastive learning ought to include. This goal supports the notion that an excellent perspective should emphasize gathering pertinent details while reducing redundancy, providing insightful information for efficient graph representation learning.

To delve deeper into the essential information of graphs, we establish a property that a good view should possess:

**Definition** **1.** *A good view is intended to be a substructure of the corresponding graph to mitigate the introduction of artificially induced noise.*

In computer vision, important information is frequently obtained via random disturbance of the data, and the generated noise is known to support robust representation learning [9]. Graph augmentation is more difficult to interpret and less intuitive than data augmentation on photos, which does not require in-depth topic expertise. It is challenging to produce high-quality contrastive samples for graphs because of this complexity [12,17]. Thus, we argue that random perturbation should not be used to introduce fake noise in a decent representation of graphs.

Given a normal graph *G* and its $G^{*}$ view, the mutual information between *G* and $G^{*}$ may be written as follows:

(5) $$I\left(G^{*};G\right)=H\left(G^{*}\right)-H\left(G^{*}|G\right),$$

where the entropy of $G^{*}$ is $H(G^{*})$, and the conditional entropy of $G^{*}$ conditioned on *G* is $H(G^{*}|G)$ (for simplicity, we ignore the graph encoder *f* without losing generality). According to the definition of Shannon entropy [14], the uncertainties of $G^{*}$ and *G* are also represented by the variables $H(G^{*})$ and H(G), respectively.

Furthermore, we may deduce that $H(G^{*}|G)=0$ since, in accordance with Definition 1, the information encoded in $G^{*}$ is a subset of the information in *G*. Thus, it is possible to reduce the mutual information to the following:

(6) $$I\left(G^{*};G\right)=H\left(G^{*}\right).$$

Consequently, to capture the essential information of the input graph, we need to minimize the uncertainty of graph *G*, expressed as $\min,\;H(G^{*})$. Here, we provide the definition of a good view for graph contrastive learning (GCL).

**Definition** **2.** *The good view of a graph should have minimal structural uncertainty.*

Shannon entropy is a useful measure for assessing structural information in graphs, but it is not appropriate for our purposes. Brooks posed the dilemma of how to characterize a graph’s underlying data in a way that makes it possible to understand its fundamental structure [15]. Shannon likewise considered whether communication graph analysis could be aided by a structural theory of information [32].

To measure the uncertainty of a graph’s structure, the notion of structural entropy was recently proposed and described on graphs [16]. This structural information theory states that a coding tree encodes a graph. The structural entropy of a graph G=(V,E) on its coding tree *T* is defined as

(7) $$H^{T}\left(G\right)=-\sum_{v_{\tau }\in T}\frac{g_{v_{\tau }}}{vol(V)}\log\frac{vol(v_{\tau })}{vol(v_{\tau }^{+})},$$

where $g_{v_{t}}$ denotes the number of edges with an endpoint in the leaf node partition of $v_{t}$, $v_{t}^{+}$ is the parent of $v_{t}$, and vol(V) and $vol(v_{t})$ are the sums of degrees of leaf nodes in V and $v_{t}$, respectively. Moreover, $v_{t}$ is a nonroot node in *T* and can also be viewed as a node subset ⊂V according to its leaf node partition in *T*.

The objective is to find the ideal coding tree *T* with the smallest entropy, or $\min_{T}H^{T}\left(G\right)$, in order to interpret the fundamental structure of graph *G* with the least amount of structural uncertainty. A coding tree with a matching constant height is preferable because real-world networks frequently have a natural structure with a defined hierarchy. Here, the ideal coding tree with a height of *k* is decoded using the *k*-dimensional structural entropy:

(8) $$H^{k}\left(G\right)=\min_{\forall T:\mathrm{Height}(T)=k}H^{T}\left(G\right).$$

#### An Instantiation of Essential Structure Decoding and Representation

In this subsection, we will initially present a practical instantiation for decoding the essential structure to minimize structural uncertainty. Following this, we will introduce a novel Graph Convolutional Network (GCN) for the coding tree representation based on Graph Neural Networks (GNNs).

We seek a method to decode the coding tree of height *k* from a given graph, guided by the notion of *k*-dimensional structural entropy. A coding tree *T* can be created for a graph G=(V,E), with $v_{r}$ serving as the tree’s root node and V serving as its leaf nodes. Two functions for the coding tree *T* are defined as follows:

**Definition** **3.** *Let T be any coding tree for the graph G=(V,E), where the leaf nodes are V and the root node is $v_{r}$. In T, let $v_{i}\in v_{r}.children$ and $v_{j}\in v_{r}.children$ be any two nodes $\left(v_{i},v_{j}\right)$. Defining a function ${\mathrm{MERGE}}_{T}\left(v_{i},v_{j}\right)$ for T that inserts a new node $v_{\varepsilon }$ between $\left(v_{i},v_{j}\right)$ and $v_{r}$*

(9) $$\begin{array}{c} v_{\varepsilon }.children\leftarrow v_{i}; \end{array}$$

(10) $$\begin{array}{c} v_{\varepsilon }.children\leftarrow v_{j}; \end{array}$$

(11) $$\begin{array}{c} v_{r}.children\leftarrow v_{\varepsilon }; \end{array}$$

**Definition** **4.** *In accordance with the configuration described in Definition 3, given any two nodes $\left(v_{i},v_{j}\right)$, where $v_{i}\in v_{j}.children$. Create the function ${delete}_{T}\left(v_{i}\right)$ for T in order to merge $v_{i}.children$ with $v_{j}.children$ and delete $v_{i}$ from T:*

(12) $$v_{j}.children\leftarrow v_{i}.children;$$

Algorithm 1 provides a greedy algorithm based on the two provided functions that computes the coding tree with a given height *k* using structural entropy minimization. More specifically, starting from the bottom, a full-height binary coding tree is created. In this step, the goal is to maximize the decrease in structural entropy by merging two child nodes of the root to produce a new division in each iteration. In the second step, we must eliminate nodes from the previous full-height binary coding tree in order to compress it to meet a set number of graph coarsenings. Each time, we take an inner-node from *T*, and after removing it, *T* has the lowest structural entropy. With the help of structural entropy, we have already created a coding tree with a certain height *k* at the conclusion of the second step. When implementing hierarchical pooling based on such a coding tree, there can be nodes that, due to cross-layer linkages, do not have an immediate successor in the following layer. This will result in nodes being missing. As a result, in order to maintain the integrity of information transfer across layers and to avoid interfering with *G*’s structural entropy on the coding tree *T*, we must complete the third step. Ultimately, $T=(V^{T},E^{T})$, $V^{T}=\left(V_{0}^{T},\ldots ,V_{k}^{T}\right)$, and $V_{0}^{T}=V$ may be used to create a coding tree *T* for the provided graph *G*. Furthermore, it is possible to obtain the cluster assignment matrices from $E^{T}$, that is, $S=(S_{1},\ldots ,S_{k})$.

Complexity analysis of Algorithm 1. With $h_{max}$ representing the height of the coding tree *T* following the first step, the runtime complexity of Algorithm 1 is $O(2n+h_{max}\left(m\log n+n\right))$. During the structural entropy reduction process, $h_{max}$ will be about logn, since coding tree *T* tends to be balanced. Algorithm 1’s runtime roughly grows linearly with the number of edges, as a network often has more edges than nodes, namely m≫n. The algorithm under consideration maintains two data structures: a coding tree and a graph. The space complexity of the algorithm is O(m+n), where *n* denotes the number of nodes and *m* represents the number of edges. Specifically, the graph requires O(n+m) space. The coding tree, on the other hand, necessitates O(n) space, given that the number of nodes in the coding tree is less than or equal to 2n.
**Algorithm 1** Coding tree with height *k* via structural entropy minimization.**Input:** a graph G=(V,E), a positive integer k>1
**Output:** a coding tree *T* with height *k*
1:Generate a coding tree *T* with a root node $v_{r}$ and all nodes in V as leaf nodes;2:// Stage 1: Bottom to top construction;3:**while**$|v_{r}.children|>2$**do**4:   Select $v_{i}$ and $v_{j}$ from $v_{r}.children$, conditioned on     $argmax_{\left(v_{i},v_{j}\right)}\left\{H^{T}\left(G\right)-H^{T_{\mathrm{MERGE}(v_{i},v_{j})}}\left(G\right)\right\}$;5:   $\mathrm{MERGE}(v_{i},v_{j})$;6:**end while**7:// Stage 2: Compress *T* to the certain height *k*;8:   **while** Height(T)>k **do**9:     Select $v_{i}$ from *T*, conditioned on     $argmin_{v_{i}}\left\{H^{T_{\mathrm{REMOVE}(v_{i})}}\left(G\right)-H^{T}\left(G\right)\right|$
                $v_{i}\neq v_{r}\;\&\;v_{i}\notin V\}$;10:     $\mathrm{REMOVE}(v_{i})$;11:**end while**12:// Stage 3: Fill *T* to avoid cross-layer links;13:**for** $v_{i}\in T$ **do**14:     **if** $|\mathrm{Height}\left(v_{i}.parent\right)-\mathrm{Height}\left(v_{i}\right)|>1$ **then**15:          insert a new node $v_{\varepsilon }$ between $v_{i}$ and $v_{j}$;16:     **end if**17:**end for**18:return *T*;


Coding tree representation learning. The coding tree functions act as a compact representation of the original graph structure, preserving its essential information while minimizing redundancy and avoiding noise introduced during augmentation. To seamlessly integrate the coding tree into the graph contrastive learning architecture, a novel encoder is introduced. In order to capture the hierarchical structure of nodes within the coding tree, a tree positional encoding mechanism is employed. This mechanism enables the model to distinguish nodes located at different depths. The positional embedding $p^{i}$ for nodes of height *i* is defined as follows:

(13) $$p^{i}=\mathrm{PositionEncoder}\left(i\right),$$

where PositionEncoder(i) generates unique embeddings for each layer of the coding tree. In practical experiments, the implementation of PositionEncoder utilizes the embedding layer provided by PyTorch.

The encoder, a novel recursive neural network, propagates information iteratively from the bottom to the top. The process begins with the leaf nodes, and as iterations progress, the model gradually learns representations for each non-leaf node by aggregating the representations of its descendants. This iterative process culminates in the derivation of the representation for the root node. We utilize a Gated Recurrent Unit (GRU) [33] as the aggregate function. Consequently, the representation of a non-leaf node with height *i* in the coding tree is computed as

(14) $$\begin{array}{cc} {\hat{r}}_{v}^{i} & =\sum_{u\in C(v)}r_{u}^{\left(i-1\right)}, \end{array}$$

(15) $$\begin{array}{cc} r_{v}^{i} & =\mathrm{GRU}\left(p^{i},\;\;\;\;{\hat{r}}_{v}^{i}\right), \end{array}$$

where $r_{v}^{i}$ is the hidden representation of node *v*, $p^{\left(i\right)}$ is taken as the input, and ${\hat{r}}_{v}^{i}$ is the aggregated information from the children of node *v*, which represents the hidden state. More specifically, $r_{v}^{i}$ is given by

(16) $$\begin{array}{cc} s_{v}^{i} & =\sigma \left(W_{s}p^{i}+U_{s}{\hat{r}}_{v}^{i}\right), \end{array}$$

(17) $$\begin{array}{cc} z_{v}^{i} & =\sigma \left(W_{z}p^{i}+U_{z}{\hat{r}}_{v}^{i}\right), \end{array}$$

(18) $$\begin{array}{cc} {\tilde{r}}_{v}^{i} & =\tanh\left(W_{r}p^{i}+U_{r}\left(s_{v}^{i}⊙{\hat{r}}_{v}^{i}\right)\right), \end{array}$$

(19) $$\begin{array}{cc} r_{v}^{i} & =\left(1-z_{v}^{i}\right)⊙{\tilde{r}}_{v}^{i}+z_{v}^{i}⊙{\hat{r}}_{v}^{i}, \end{array}$$

where σ is the logistic sigmoid function, ⊙ denotes element-wise multiplication, $W_{*}$ and $U_{*}$ refer to weight matrices used for linear transformations of vectors that control how the input and hidden state are combined to produce the new state $r_{v}^{i}$.

Complexity analysis of coding tree learning. The runtime complexity is $O(nd^{2})$. This process involves a propagation step, which traverses the tree from the leaf nodes to the root, taking O(n) time. At each node, the GRU executes update and reset operations, both of which involve matrix multiplications. Assuming the dimension of the hidden feature to be *d*, these operations require $O(d^{2})$ time. The space complexity of the learning process is $O(nd+d^{2})$, determined by the storage of the node features and the parameters of the GRU. Each node in the tree possesses a *d*-dimensional feature vector, hence storing the features for all tree nodes requires O(nd) space. The GRU parameters, which are of size $O(d^{2})$, also contribute to the space complexity.

For the contrastive loss calculation, $x_{\mathrm{root}}^{k}$ (i.e., the feature vector of the root node) can be used to represent the entire coding tree. However, recognizing the distinct functionality of the natural hierarchy, we incorporate the embedded information from each iteration through skip connections. Specifically, we learn the coding tree with concatenated layer representations:

(20) $$r_{T}=\left[\mathrm{POOL}\left(\left\{r_{v}^{0}|v\in V_{T}^{0}\right\}\right)\;\;;\;\;\mathrm{POOL}\left(\left\{r_{v}^{1}|v\in V_{T}^{1}\right\}\right)\;\;;\;\;\ldots \;\;;\;\;x_{root}^{k}\right)],$$

where $r_{v}^{i}$ is the hidden representation and *k* is the height of tree *T*. In particular, POOL in Equation (20) will be implemented by the widely used pooling approaches, such as summation or averaging.

## 4. Experiment Setup

In this section, we dedicate ourselves to evaluating CtrlGCL through extensive experiments. We begin by describing the experimental setup for graph classification, covering both semi-supervised and unsupervised learning scenarios. Subsequently, we validate the effectiveness of the proposed good view against state-of-the-art (SOTA) competitors, contrasting with pre-defined rules for graph augmentation.

Given that our good view is orthogonal to previous works on graph augmentations, we conduct additional analyses to demonstrate the collaborative capabilities of CtrlGCL with existing approaches. This comprehensive evaluation aims to showcase the versatility and effectiveness of CtrlGCL across various experimental settings and in collaboration with diverse graph augmentation strategies.

### 4.1. Datasets

Various benchmarks for view validation are adopted from TUDatasets [34]. Specifically, we utilized six datasets for social networks, including IMDB-BINARY, IMDB-MULTI, COLLAB, REDDIT-MULTI-5K, REDDIT-BINARY, and GITHUB; two datasets for small molecules, including NCI1 and MUTAG; and two datasets for bioinformatics, including PROTEINS and DD.

We performed trials for several graph property prediction tasks on a large variety of datasets from different disciplines. We offer thorough explanations of each of the ten benchmark datasets utilized in this investigation. The statistics for these datasets are summarized in Table 1.

Social Network. IMDB-BINARY and -MULTI are products of a movie set working together. Actors or actresses are represented as nodes in these two databases, while their collaboration in a certain film is represented by edges. Every graph has a label that relates to the genre of the particular film that it is connected to. Comparably, COLLAB is a scientific domain collaboration dataset made up of three public collaboration datasets: condensed matter physics, high-energy physics, and astronomy. For the graphs in this benchmark, researchers from different fields have created different ego networks. The study field that each graph’s nodes belong to is indicated by its label. The balanced datasets REDDIT-BINARY or -MULTTI-5K have graphs that each represent an online discussion, and the nodes stand for users. If two nodes reply to one other’s comments, then an edge is formed between them. Sorting each graph into the appropriate community or subreddit is the current work at hand.

Bioinformatics. Protein structure diagrams are included in DD. Every node symbolizes an amino acid, and edges arise when two nodes are separated by less than $6\;A^{\circ }$. If a protein is an enzyme or not, it is indicated on the label. A dataset known as PROTEINS has secondary structural elements (SSEs) as its nodes. If two nodes are next to one another in a 3D space or in the provided amino acid sequence, then an edge exists between them. Three discrete labels that stand for helices, sheets, or turns are found in the dataset. The NCI1 dataset comes from the field of chemical informatics, where each input graph is used as a representation of a compound: each vertex represents an atom of a molecule, and the edges between vertices represent bonds between atoms. This dataset is related to anti-cancer screening, where chemicals are evaluated as positive or negative for cellular lung cancer. A total of 37 distinct labels make up this dataset. Seven different graph types are found in MUTAG, which are formed from 188 different carcinogenic aromatic and heteroaromatic nitro chemicals. Ten datasets were used, and their characteristics are compiled in Table 1.

### 4.2. Configuration

Our two-block contrastive learning framework with the decoded basic graph structure is illustrated in Figure 1. In the block pertaining to graph augmentations, we utilize the identical GNN architectures with their original hyper-parameters under different experiment circumstances, as per the methodology used in GraphCL (the first technique for graph contrastive learning) [7]. To be more precise, we utilized GIN with 32 hidden units and 3 layers for unsupervised representation learning and ResGCN with 128 hidden units and 5 layers for semi-supervised learning. Furthermore, graphs with a default augmentation strength of 0.2 were subjected to the same data augmentations.

The number of tree encoder layers for coding tree representation learning was determined by the tree height, which ranges from two to five. There were two layers in each iteration of the MLP (Multilayer Perceptron). In order to preserve uniformity with GraphCL across the different experiment configurations, the encoder’s hidden dimensions are specified with the corresponding setting. The optimal hyper-parameter combination was determined based on the performance on the validation sets.

Semi-Supervised Learning. We performed five trials for each dataset, with a 10% label rate, meaning that each experiment corresponds to a 10-fold assessment, as described in [7]. For every experiment, we present the accuracy (%) mean, and standard deviation. A grid search was used to set the epoch number to {20,40,60,80,100} and the learning rate to {0.01,0.001,0.0001} for pre-training. We fine-tuned using the same parameters as described in [7]: learning rate of 0.001, batch size of 128, hidden dimension of 128, and 100 epochs of training for the pre-trained models.

Unsupervised Learning. Each experiment was carried out five times, and as shown in [20], each experiment corresponds to a 10-fold assessment. For every experiment, we present the accuracy (%) mean, and standard deviation. Models were tested every 10 epochs and trained for 20 epochs in order to learn the graph representations. The batch size was 128 and the hidden dimension was 32.

Data Augmentations on Graphs. There are four common categories of data augmentations for graph-structured data, which correspond to the data augmentations utilized in GraphCL [7].

Edge Perturbation. Here, the connectivities in *G* are disturbed by arbitrarily adding or removing a specific percentage of edges. This operation is predicated on the notion that the semantic meaning of *G* is relatively resilient to variations in edge connection patterns. To add or remove each edge, we further adhered to an independent, identically distributed (i.i.d.) uniform distribution.

Node Dropping. Node dropping, given the graph *G*, randomly discards a subset of vertices and their connections. This procedure makes the assumption that the semantic meaning of *G* is unaffected by the missing portion of vertices. The default independent and identically distributed uniform distribution governs each node’s dropping probability.

Attribute Masking. With attribute masking, models are prompted to retrieve masked vertex attributes by utilizing their context, that is, the remaining attributes. The underlying premise of this operation is that the model’s predictions are not substantially impacted by the missing partial vertex information.

Subgraphs. Using a random walk, this procedure samples a subgraph from *G*. It makes the assumption that *G*’s partial local structure can effectively retain its semantics.

### 4.3. Learning Protocols

We employed the necessary learning techniques to enable fair comparison with state-of-the-art (SOTA) efforts. All data were utilized for model pre-training in unsupervised representation learning [20], after which the learnt graph embeddings were fed into an SVM classifier for 10-fold cross-validation. Two learning settings were used for semi-supervised learning [7]. Only the training dataset was used for pre-training when the datasets had a public training/validation/test split. Ten percent of the training data was used for fine-tuning, and the validation/test sets yielded the final assessment findings. All samples were used for pre-training on datasets without these splits, and assessment and fine-tuning were carried out across ten assessments.

### 4.4. The Compared Methods

In the two-block design of CtrlGCL, there were three types of results: (1) only graph embedding, termed CtrlGCL-G; (2) only coding tree embedding, termed CtrlGCL-T; (3) and the hybrid of graph embedding and coding tree embedding, termed CtrlGCL-H.

In unsupervised learning, we adopted eight baselines that fall into three categories. We used the published hyper-parameters of these methods. The first set included three state-of-the-art (SOTA) kernel-based methods: GL [35], WL [36], and DGK [37]. The second set comprised four heuristic self-supervised methods: node2vec [38], sub2vec [39], graph2vec [40], and InfoGraph [20]. GraphCL, the last technique in this group, uses the same pre-established augmentation rules on graphs for unsupervised learning [7]. The default augmentation ratio was 0.2 (dropping, perturbation, masking, and subgraph). In addition to the individual use of particular data augmentation, GraphCL uses augmentation pools for contrastive learning. Specifically, biological molecules are treated using node dropping and subgraphs; all augmentations are applied to dense social networks; and for sparse social networks, all except attribute masking are employed.

Under semi-supervised learning, we considered five baselines:

(1) A naive GCN without pre-training [7], which is directly trained with 10% labeled data from random initialization.
(2) GAE [41], a predictive method based on edge-based reconstruction in the pre-training phase.
(3) Infomax [6], a node-embedding method with global–local representation consistency.
(4) ContextPred [19], a method using sub-structure information preserving.
(5) GraphCL [7], the first graph contrastive learning method with data augmentations.

## 5. Results

### 5.1. Unsupervised Learning

In the context of unsupervised learning, Table 2 summarizes the classification accuracy of CtrlGCL and the comparative approaches. When considering the baselines, the results show a notable boost in performance with the addition of a good view. When the last column for average rank is taken into account, the three CtrlGCL variations have the highest ranks. Significantly, our techniques outperform the competing approaches on all seven benchmarks, with the exception of NCI1. In addition, in the case without kernel-based techniques, CtrlGCL-G consistently achieves the maximum accuracy. Our findings indicate that, in unsupervised learning scenarios, our techniques regularly beat the most advanced approaches.

In addition to the overall superior performance of CtrlGCL, we delved deeper into the specific performance of each variant. As indicated by the marker ^*^ in Table 2, we identified the best performances among the three variants. Among the four datasets in social networks, CtrlGCL-T achieves the highest accuracies on COLLAB and IMDB-BINARY, while CtrlGCL-H outperforms on the REDDIT datasets. Notably, the edge density (average edges divided by average nodes) of COLLAB and IMDB-BINARY is much higher than that of the REDDIT datasets, suggesting higher structural uncertainty in COLLAB and IMDB-BINARY. Therefore, the performance of CtrlGCL-T/H on social networks confirms the effectiveness of the proposed good view in minimizing structural uncertainty. Additionally, for datasets with higher structural uncertainty, the proposed good view provides high-quality graph representation, while for datasets with lower structural uncertainty, the proposed method presents sufficient information for performance improvement. In bioinformatics datasets, a similar phenomenon can be observed on the PROTEINS dataset, which also has higher edge density. However, different results are shown in DD, where the non-structural properties of this type of protein may explain the variation. For the other two datasets with lower edge density, CtrlGCL-G, which employs graph embedding, shows the best performance, implying lower structural uncertainty in these two sets.

### 5.2. Semi-Supervised Learning

Under semi-supervised learning, the accuracies of our models and the compared methods are presented in Table 3. Notably, GtrlGCL is better than these state-of-the-art (SOTA) methods across all benchmarks. With the exception of CtrlGCL-T’s poor performance, CtrlGCL-G and CtrlGCL-H rank as the top two methods overall. Specifically, CtrlGCL-G achieves the highest accuracy on three out of the seven benchmarks, while CtrlGCL-H holds this position in four out of the seven benchmarks. These results demonstrate the effectiveness of our methods under semi-supervised learning. The results from Table 3 show that GtrlGCL-H, which incorporates both graph embedding and coding tree embedding, achieves the highest mean rank and significant performance improvement compared to CtrlGCL-G. This validates the effectiveness of our proposed good view in semi-supervised learning. However, the poor performance of GtrlGCL-T is a concern. Nonetheless, the overall results demonstrate the value of our methods in semi-supervised learning, with CtrlGCL-G and CtrlGCL-H ranking highly.

### 5.3. Orthogonal to Graph Augmentations

In this section, we evaluate the collaborative potential of CtrlGCL by integrating it with four established graph augmentation techniques: AD-GCL [10], JOAO [11], AutoGCL [42], and RGCL [8]. These approaches introduce innovative strategies for generating augmented views, and we explore their synergy with CtrlGCL in an unsupervised learning setting.

AD-GCL [10]. We used the same configurations as before in the cooperative experiment with AD-GCL, and we swapped out its anchor view with the proposed good view from CtrlGCL. The approaches were assessed using a linear classifier after they had been trained using the relevant self-supervised goal. We adhered to the linear assessment methodology presented in AD-GCL [10]. Specifically, once the encoder provides representations, a Logistic (+L2) classifier is trained on top and evaluated for classification tasks. The classifier was implemented using Scikit-learn [43] or LibLinear [44] solvers. Finally, the lone hyper-parameter of the downstream linear model, that is, the L2 regularization strength, is grid searched among {0.001, 0.01, 0.1, 1, 10, 100, 1000} on the validation set for every single representation evaluation. Accuracy (%) was selected as the test parameter in accordance with the usual procedure. Every AD-GCL experiment was ran ten times using a different set of random seeds. For every dataset, we provided the mean and standard deviation of the associated test measure.

The encoder utilized in the joint experiment with AD-GCL was the GIN encoder [45]. To guarantee a fair comparison, the encoder was fixed and not adjusted while performing self-supervised learning (i.e., embedding dimension, number of layers, pooling type) for all the approaches. This decision was made with the intention of completely attributing any performance disparity to the self-supervised goal and excluding the encoder design. The GIN encoder was configured with the following unique hyper-parameters: a batch size of 32, a hidden dimension of 32, five GIN layers, summation as the graph readout function, and a dropout set at 0.5. Adam was used for optimization, and the learning rates in AD-GCL were adjusted to be within {0.01,0.005,0.001} for both the encoder and the augmenter. Since asymmetric learning rates for the augmenter and encoder tend to render the training non-stable, the learning rate was set to 0.001 for all datasets and experiments were carried out to ensure stability [10]. Using the validation set, the number of training epochs was selected as {20,50,80,100,150}.

JOAO(v2) [11]. We used the same experimental setup as the original study in our collaboration with JOAO, but we made the following significant change: we swapped out one of the two views with the “good view” suggested in CtrlGCL. We were able to assess the effectiveness of our suggested view selection technique as a result. In this experiment, GIN was also adopted as the basic graph encoder [45], while non-linear SVM was employed for evaluation as GraphCL. To strike a compromise between the contrastive loss and view distance, the hyper-parameter γ introduced in JOAO was adjusted within the range {0.01,0.1,1}. Notably, because multiple projection heads were used, JOAOv2 was pre-trained twice as many epochs than JOAO, despite our 20 epochs of pre-training JOAO. With this modification, we were able to evaluate the relative performance of the two approaches and make a direct comparison.

AutoGCL [42]. We adopted the naive training strategy proposed in AutoGCL to make a fair comparison. Specifically, we retained one of the two graph generators and assigned our proposed anchor view to the blank position. In particular, AutoGCL extends the layer number of the graph encoder from 3 to 5 and the hidden size from 32 to 128. Moreover, AutoGCL was pre-trained with 30 epochs rather than 20 epochs.

RGCL [8]. In cooperation with RGCL [8], we faithfully followed the experiment settings revealed in their codes while replacing one of the two rationale-augmented views with SEGA. Note that, the tuned hyper-parameters in RGCL include the learning rate, sampling ratio ρ, loss temperature τ, and loss balance λ. In particular, RGCL was pre-trained on 40 epochs in total and evaluated every 5 epochs.

The unsupervised learning classification accuracies (%) of CtrlGCL in collaboration with the four methods for augmentations are presented in Table 4. The last column displays the average accuracies (%) over all datasets, and the three versions of CtrlGCL always suppress their corresponding partner view, highlighting the efficacy of the proposed good view in minimizing structural uncertainty. Specifically, when combined with AD-GCL-FIX, CtrlGCL-T achieved the highest accuracies on three out of the nine datasets, while CtrlGCL-H outperformed on the remaining six benchmarks. Despite a few setbacks in the collaboration with JOAO, the overall superior performance underscores the success of CtrlGCL in these extensive experiments. Furthermore, when paired with AutoGCL, CtrlGCL-T attained the highest accuracies on two out of the eight datasets, while CtrlGCL-H excelled on five out of the eight benchmarks. In the case of RGCL, the collaboration yielded the highest results on all eight datasets. To elaborate, CtrlGCL-G outperformed on the MUTAG dataset, CtrlGCL-T excelled on the RED-B and IMDB-B datasets, and CtrlGCL-H achieved the best results on the remaining five datasets.

### 5.4. Memory Efficiency

**Figure 2:**
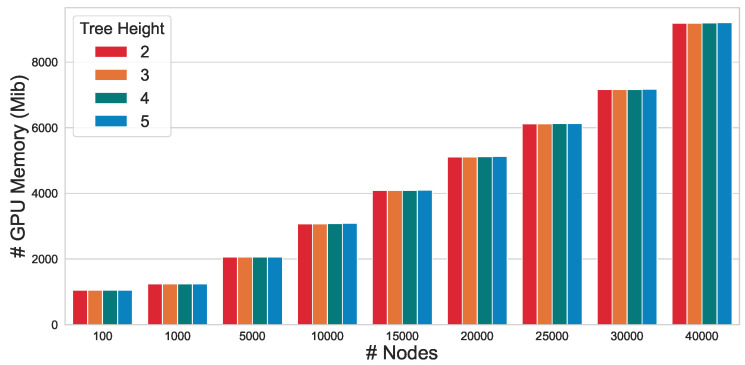
GPU memory efficiency of CtrlGCL with varying input graph sizes.

To evaluate the scalability of the proposed approach, we conducted an in-depth analysis of the GPU memory efficiency of CtrlGCL on Erdos–Renyi graphs [46]. In line with the methodology employed in prior research [47], we generated Erdos–Renyi graphs by modulating the number of nodes *n* while maintaining the edge size *m* at twice the number of nodes m=2n. As depicted in Figure 2, our CtrlGCL demonstrates high memory efficiency, attributable to the computational efficiency of the tree encoder. This characteristic renders it particularly practical for large-scale graph applications. Notably, a comparison of memory usage across different tree heights with the same graph size reveals that the GPU memory consumption remains relatively constant, further underscoring the scalability of our proposed approach.

## 6. Conclusions

In this study, our focus is on exploring a good view for graph contrastive learning. Upon leveraging insights from the structural information and graph information bottleneck theory, we proposed the definition that a good view should possess minimal structural uncertainty for a graph. Taking this concept further, we introduced CtrlGCL, a practical implementation for graph contrastive learning through coding tree representations. Our approach utilized an optimization algorithm driven by structural entropy to approximate the minimization of structural uncertainty, resulting in coding trees that encapsulate essential graph information. The encoder, designed with the convolution mechanism of GNNs, was tailored for learning representations from coding trees. The effectiveness of our proposed approach was extensively validated across various benchmarks in both unsupervised and semi-supervised learning. This validation was reflected in the average ranking and average accuracy, demonstrating superior performance compared to other state-of-the-art methods. Specifically, our approach, implemented via GtrlGCL-H, yielded an average accuracy enhancement of 1.06% in the context of unsupervised learning when compared to GraphCL. In the semi-supervised learning scenario, CtrlGCL-G outperformed GraphCL, with an increase of 0.22%. Notably, in orthogonal experiments, almost all versions of CtrlGCL-H surpassed the corresponding baselines by more than 1% for average accuracy under unsupervised learning.

Despite the superiority of the proposed “good view” for graph contrastive learning based on structural entropy, the current definition of structural entropy only considers the structural information. This limitation may affect tasks such as node classification and link prediction that heavily rely on node features, potentially limiting the benefits of the proposed good view. Our future research direction entails enhancing our methods through the refinement of the structural entropy theory or by exploring the amalgamation of multiple entropy measures. The emphasis on minimizing uncertainty in node features suggests promising avenues for future research, exploration, and improvement. We look forward to continuing our work in this exciting field.

## Figures and Tables

**Table 1:** Statistics for the datasets from TUDataset.

Dataset	#Graphs	#Classes	Avg. #Nodes	Avg. #Edges
REDDIT-BINARY	2000	2	429.63	497.75
COLLAB	5000	3	74.49	2457.78
REDDIT-MULTI-5K	4999	5	508.52	594.87
IMDB-MULTI	1500	3	13.00	65.94
IMDB-BINARY	1000	2	19.77	96.53
GITHUB	12,725	2	113.79	234.64
MUTAG	188	2	17.93	19.79
NCI1	4110	2	29.87	32.30
DD	1178	2	284.32	715.66
PROTEINS	1113	2	39.06	72.82

**Table 2:** The average classification accuracies (%), along with their standard deviations (±Std.), obtained from five separate runs of the compared methods via unsupervised representation learning. The **bold** text highlights the best overall performances among all methods. The marker ^*^ indicates the best performance among the three variations of CtrlGCL. The term A.R. stands for average rank, which is used to assess the relative performance of each method. The results for the baselines were obtained from previously published works.

	NCI1	PROTEINS	DD	MUTAG	COLLAB	RED-B	RED-M5K	IMDB-B	A.R.
Avg. #Nodes	29.87	39.06	284.32	17.93	74.49	429.63	508.52	19.77	
Avg. #Edges	32.30	72.82	715.66	19.79	2457.78	497.75	594.87	86.53	
GL	-	-	-	81.66 ± 2.11	-	77.34 ± 0.18	41.01 ± 0.17	65.87 ± 0.98	8.3
WL	80.01 ± 0.50	72.92 ± 0.56	-	80.72 ± 3.00	-	68.82 ± 0.41	46.06 ± 0.21	72.30 ± 3.44	6.7
DGK	**80.31 ± 0.46**	73.30 ± 0.82	-	87.44 ± 2.72	-	78.04 ± 0.39	41.27 ± 0.18	66.96 ± 0.56	5.7
node2vec	54.89 ± 1.61	57.49 ± 3.57	-	72.63 ± 10.20	-	-	-	-	9.3
sub2vec	52.84 ± 1.47	53.03 ± 5.55	-	61.05 ± 15.80	-	71.48 ± 0.41	36.69 ± 0.42	55.26 ± 1.54	10
graph2vec	73.22 ± 1.81	73.30 ± 2.05	-	83.15 ± 9.25	-	75.78 ± 1.03	47.86 ± 0.26	71.10 ± 0.54	7.0
InfoGraph	76.20 ± 1.06	74.44 ± 0.31	72.85 ± 1.78	89.01 ± 1.13	70.65 ± 1.13	82.50 ± 1.42	53.46 ± 1.03	73.03 ± 0.87	4.3
GraphCL	77.87 ± 0.41	74.39 ± 0.45	78.62 ± 0.40	86.80 ± 1.34	71.36 ± 1.15	89.53 ± 0.84	55.99 ± 0.28	71.14 ± 0.44	4.0
CtrlGCL-G	79.00 ± 0.72^*^	75.79 ± 0.27	78.15 ± 0.56	90.21 ± 0.66 *	70.73 ± 0.65	89.85 ± 0.56	55.27 ± 0.32	72.30 ± 0.24	2.9
CtrlGCL-T	74.92 ± 0.53	76.01 ± 0.42 *	77.34 ± 1.03	88.50 ± 1.30	74.12 ± 0.47 *	88.67 ± 0.60	52.26 ± 0.69	73.58 ± 0.44 *	3.4
CtrlGCL-H	78.86 ± 0.38	75.85 ± 0.46	78.76 ± 0.57 *	90.17 ± 0.97	71.44 ± 0.45	90.21 ± 0.65 *	56.13 ± 0.30 *	72.78 ± 0.64	2.0

**Table 3:** The comparison approaches’ average accuracies (%) and standard deviations (±Std) during semi-supervised learning with 10% labels. The strategy that performed the best overall is highlighted in the **bold** text. Average rank, or A.R., is used to evaluate the relative effectiveness of each approach. The baseline results are from previously released publications.

	NCI1	PROTEINS	DD	COLLAB	RED-B	RED-M5K	GITHUB	A.R.
No Pre-Train	73.72 ± 0.24	70.40 ± 1.51	73.56 ± 0.41	73.71 ± 0.27	86.63 ± 0.27	51.33 ± 0.44	60.87 ± 0.17	7.0
GAE	74.36 ± 0.24	70.51 ± 0.17	74.54 ± 0.68	75.09 ± 0.19	87.69 ± 0.40	53.58 ± 0.13	63.89 ± 0.52	5.0
Infomax	74.86 ± 0.26	72.27 ± 0.40	75.78 ± 0.34	73.76 ± 0.29	88.66 ± 0.95	53.61 ± 0.31	65.21 ± 0.88	4.0
ContextPred	73.00 ± 0.30	70.23 ± 0.63	74.66 ± 0.51	73.69 ± 0.37	84.76 ± 0.52	51.23 ± 0.84	62.35 ± 0.73	7.3
GraphCL	74.63 ± 0.25	74.17 ± 0.34	76.17 ± 1.37	74.23 ± 0.21	89.11 ± 0.19	52.55 ± 0.45	65.81 ± 0.79	3.3
CtrlGCL-G	74.72 ± 0.26	**74.65 ± 0.54**	**76.33 ± 0.43**	74.26 ± 0.27	**89.40 ± 0.23**	52.93 ± 0.37	65.92 ± 0.64	2.1
CtrlGCL-T	71.80 ± 0.35	73.31 ± 0.47	75.63 ± 0.58	73.36 ± 0.35	88.70 ± 0.15	52.11 ± 0.34	65.39 ± 0.58	5.6
CtrlGCL-H	**75.09 ± 0.22**	73.85 ± 0.53	75.82 ± 0.65	**75.18 ± 0.22**	89.35 ± 0.27	**53.73 ± 0.28**	**66.01 ± 0.66**	1.7

**Table 4:** The average accuracy (%) ± standard deviation (over five times) of various methods used in unsupervised learning. Boldface type highlights the best performances for each individual dataset. A.A. signifies the average accuracy across all datasets. The results of AD-GCL-FIX, JOAO(v2), AutoGCL, and RGCL were obtained from their respective papers.

View1	View2	NCI1	PROTEINS	DD	MUTAG	COLLAB	RED-B	RED-M5K	IMDB-B	IMDB-M	A.A.
AD-GCL-FIX		69.57 ± 0.51	73.59 ± 0.65	74.49 ± 0.52	89.25 ± 1.45	73.71 ± 0.27	85.52 ± 0.79	53.00 ± 0.82	71.57 ± 1.01	49.04 ± 0.53	71.05
CtrlGCL-G	AD-GCL-Fix	69.93 ± 0.73	73.76 ± 0.57	74.62 ± 0.43	89.63 ± 1.54	73.77 ± 0.56	85.75 ± 0.67	53.88 ± 0.63	71.76 ± 0.57	49.44 ± 0.98	71.39
CtrlGCL-T		69.78 ± 0.32	**74.61 ± 0.81**	75.55 ± 0.51	88.20 ± 1.20	73.97 ± 0.55	86.73 ± 0.55	53.52 ± 0.31	**72.32 ± 0.49**	**50.83 ± 0.34**	71.72
CtrlGCL-H		**70.38 ± 0.76**	74.57 ± 0.50	**75.84 ± 0.64**	**89.89 ± 0.69**	**75.03 ± 0.36**	**87.74 ± 0.39**	**54.29 ± 0.54**	72.28 ± 1.40	50.03 ± 0.81	72.23
JOAO		**78.07 ± 0.47**	74.55 ± 0.41	77.32 ± 0.54	87.35 ± 1.02	69.50 ± 0.36	85.29 ± 1.35	55.74 ± 0.63	70.21 ± 3.08		74.75
CtrlGCL-G	JOAO	75.99 ± 0.59	74.95 ± 0.42	77.70 ± 0.85	87.12 ± 2.46	69.58 ± 0.27	86.57 ± 1.22	54.69 ± 0.73	71.46 ± 0.17		74.88
CtrlGCL-T		73.32 ± 0.37	**75.44 ± 0.54**	76.29 ± 0.55	85.13 ± 1.79	**72.82 ± 0.35**	86.09 ± 0.94	54.63 ± 0.64	**71.74 ± 1.26**		74.43
CtrlGCL-H		76.19 ± 0.77	75.18 ± 0.63	**78.27 ± 1.32**	**87.70 ± 1.31**	71.80 ± 0.33	**86.79 ± 1.31**	**56.17 ± 0.67**	71.66 ± 0.42		**75.47**
JOAOv2		**78.36 ± 0.53**	74.07 ± 1.10	77.40 ± 1.15	87.67 ± 0.79	69.33 ± 0.34	86.42 ± 1.45	56.03 ± 0.27	70.83 ± 0.25		75.01
CtrlGCL-G	JOAOv2	77.81 ± 0.73	75.22 ± 0.94	77.84 ± 0.84	87.82 ± 2.17	69.34 ± 0.31	87.97 ± 0.80	56.11 ± 0.33	71.68 ± 0.74		75.47
CtrlGCL-T		77.37 ± 0.31	**75.94 ± 0.88**	77.67 ± 0.48	86.77 ± 1.08	**72.76 ± 0.27**	87.32 ± 0.60	55.49 ± 0.32	**72.52 ± 0.79**		75.73
CtrlGCL-H		78.04 ± 0.19	75.25 ± 0.41	**78.37 ± 1.26**	**88.53 ± 2.45**	70.18 ± 0.34	**87.98 ± 0.29**	**56.15 ± 0.29**	72.50 ± 0.94		**75.87**
AutoGCL		**82.00 ± 0.29**	75.80 ± 0.36	77.57 ± 0.60	88.64 ± 1.08	70.12 ± 0.68	88.58 ± 1.49	56.75 ± 0.18	73.30 ± 0.40		76.60
CtrlGCL-G	AutoGCL	81.77 ± 0.32	75.63 ± 0.77	77.94 ± 0.85	88.84 ± 1.34	71.98 ± 0.83	88.75 ± 1.03	56.93 ± 0.28	73.87 ± 0.68		76.93
CtrlGCL-T		81.04 ± 0.41	**76.43 ± 0.67**	78.29 ± 0.59	88.63 ± 1.29	72.49 ± 0.47	89.59 ± 1.48	57.27 ± 0.75	**73.95 ± 0.87**		77.21
CtrlGCL-H		81.84 ± 0.53	76.38 ± 0.54	**78.31 ± 1.37**	**89.03 ± 1.01**	**72.68 ± 0.23**	**89.88 ± 1.21**	**57.43 ± 0.37**	73.94 ± 0.99		**77.44**
RGCL		78.14 ± 1.08	75.03 ± 0.43	78.86 ± 0.48	87.66 ± 1.01	70.92 ± 0.65	90.34 ± 0.58	56.38 ± 0.40	71.85 ± 0.84		76.15
CtrlGCL-G	RGCL	79.28 ± 0.94	75.28 ± 0.72	79.45 ± 0.63	**88.87 ± 1.46**	72.73 ± 0.55	90.47 ± 0.77	56.58 ± 0.41	72.19 ± 0.67		76.86
CtrlGCL-T		78.95 ± 1.53	75.87 ± 0.45	79.12 ± 0.88	87.50 ± 1.75	73.11 ± 0.24	**90.90 ± 0.62**	56.82 ± 0.29	**72.75 ± 0.66**		76.88
CtrlGCL-H		**79.42 ± 0.82**	**76.21 ± 0.46**	**79.54 ± 1.14**	88.79 ± 1.87	**73.14 ± 0.37**	90.75 ± 0.84	**57.28 ± 0.42**	72.61 ± 0.94		**77.22**

## Data Availability

Data is contained within the article.
